# Laser Soldering of Cartilage Tissue to Collagenous Biomaterial (an *in vitro* Study)

**DOI:** 10.17691/stm2023.15.6.04

**Published:** 2023-12-27

**Authors:** N.Yu. Ignatieva, O.L. Zakharkina, A.P. Sviridov

**Affiliations:** DSc, Associate Professor, Faculty of Chemistry; Lomonosov Moscow State University, 1 Leninskiye Gory, Moscow, 119991, Russia; National Research University Higher School of Economics, 20 Myasnitskaya St., Moscow, 101000, Russia; MSc, Researcher; Institute of Photonic Technologies of Federal Scientific Research Center “Crystallography and Photonics” of the Russian Academy of Sciences, 2 Pionerskaya St., Moscow, Troitsk, 108840, Russia; DSc, Leading Researcher; Institute of Photonic Technologies of Federal Scientific Research Center “Crystallography and Photonics” of the Russian Academy of Sciences, 2 Pionerskaya St., Moscow, Troitsk, 108840, Russia

**Keywords:** laser soldering, cartilage tissue, collagen denaturation

## Abstract

**The aim of the study** was to assess the effectiveness of laser soldering of biological tissues and materials for bulk heating of albumin solder on the joints of the intact and chemically modified cartilage of the porcine nasal septum.

**Materials and Methods:**

The materials for soldering were double-trypsinized and glyceraldehyde-treated plates made from cartilage of the porcine nasal septum, and intact cartilage. A 25% albumin solution was used as a solder. The junction was heated by laser radiation with the wavelengths of 1.56 and 1.68 μm through an optical fiber. The process was monitored using a digital USB microscope. After the materials were soldered, mechanical tests of the samples were conducted, and the fraction of intact collagen in the areas adjacent to the solder was determined. A thermal imager was used to record the dynamics of the temperature field in the area of laser exposure.

**Results:**

The effective soldering of cartilage tissue with collagenous biomaterial occurs with sequential application and laser heating of two/three layers of solder for radiation with wavelengths of 1.68/1.56 μm, respectively. The laser power densities for the solder layers were 0.7/0.8 W/mm^2^ (the average surface temperature ~85°C) for λ=1.68 μm and 1.77/1.34/0.96 W/mm^2^ (the average surface temperature ~100°C) for λ=1.56 μm. The tensile strength of the soldered samples reached ~12% for λ=1.56 μm and ~15% for λ=1.68 μm of the tensile strength of intact cartilage. In the tissue areas adjacent to the first layer of albumin, at a thickness of ~300 μm, most of the collagen network was destroyed. In other areas, collagen was predominantly preserved.

**Conclusion:**

Laser soldering of chemically modified and intact cartilages can be effectively conducted using radiation of λ=1.56 μm and λ=1.68 μm, absorbed not only by the solder, but also by the tissue. However, to minimize the area of degradation, it is necessary to match the diameter of the laser spot and the size of the solder-filled cavity between the construction and the intact cartilage.

## Introduction

The use of such a promising sutureless method for joining damaged collagenous tissues as the laser tissue soldering in clinical practice has been discussed for more than 25 years [1–4]. This technique involves applying solder (a concentrated albumin solution, often with a chromophore sensitizer added) to the tissue junction and treating the applied layer with laser radiation. If lasers with the wavelength of 0.8– 1.0 μm are used as the radiation source, organic dyes are used as a chromophore [1–7]. To increase the soldering strength, one can add carbon nanotubes to the solder material [6, 7]. The effectiveness of the procedure *in vivo* was confirmed with the rabbit skin laser soldering [6]. One of the major advantages of using chromophores in solder is the lack of radiation absorption by the tissue. However, due to such characteristics of laser exposure as its duration (~60 s) and a significant diameter of the laser spot (~5 mm), tissue heating occurs through the heat dissipation from the area of direct irradiation.

Radiation with the wavelength of over 1.3 μm can be absorbed by water in line with the bands position in the absorption spectrum [8], at that the chromophore is not included into the solder and the exposure time is shorter (~10 s) [9]. In some publications, laser soldering was conducted at λ=10.6 μm [10–12] and λ=1.9 μm [9, 13–15]. It should be noted that the use of radiation with λ=10.6 μm results in heating of the layer by ~30 μm [8], and the tissue edges soldering goes only on the surface, while the tissue adjacent to the solder significantly degrades, up to carbonization [12]. The radiation at λ=1.9 μm penetrates a 25% albumin solution and the tissue at ~0.2 mm [8], which provides a greater heat penetration. However, as shown by the example of the porcine cornea soldering *in vivo*, the tissue edge degradation is also seen here [9]. One should note that laser soldering with the radiation exposure of λ=2.09 μm and a penetration depth close to that of λ=1.9 μm [8] was used in clinical practice to solder authentic tissue/titanium prosthesis and auditory ossicle for the restored soundconducting system elements [16]. The authors [16] proved that laser soldering of biological tissues increased the reliability of fixation of the middle ear soundconducting system elements in case of ossiculoplasty.

Laser soldering of cartilage tissue was conducted on *in vitro* models for autografts of articular cartilage and tracheal cartilage using radiation with λ=0.808 μm [5] and λ=1.9 μm [14], respectively. The authors of the publications showed that, in case of the solder application only from the side of the laser irradiation surface, the solder remained on the surface. A more effective fixation requires filling and hardening of the solder along the entire depth of the connecting edges of the tissue, which is only possible on a substrate [5].

Scaffold fixation is an important task in the tissue engineering dealing with osteochondral defect replacement. In paper [17], the authors offered using a tissue-engineered construction based on decellularized cartilage for that purpose. This construction has a height of 2 mm, and its matrix is less thermally stable than that of the cartilage tissue. To achieve laser soldering of the scaffold to cartilage, specific exposure conditions must be met. On the one hand, the solder must fill the entire contact area between the construction and the cartilage, whereas the radiation must penetrate to the entire depth of the solder. On the other hand, damage to the construction matrix should be as minimal as possible.

**The aim of the study** was to assess the effectiveness of laser soldering using radiation with wavelengths of 1.56 and 1.68 μm for local bulk heating of the solder illustrated by soldering chemically modified cartilage (an analogue of a tissue-engineered construction) and cartilage tissue subject to minimization of the collagen matrix degradation.

## Materials and Methods

### Materials

Hyaline cartilage plates made from the porcine nasal septum were used as the basic materials. Part of the material was used to make analogs of tissueengineered constructions (ATCs). The technique for preparing ATCs from the basic cartilage is described in detail in [17] and included all stages except for the targeted laser exposure. Stabilization was conducted in a 0.02 M glyceraldehyde solution for 54 h.

**Figure 1.**
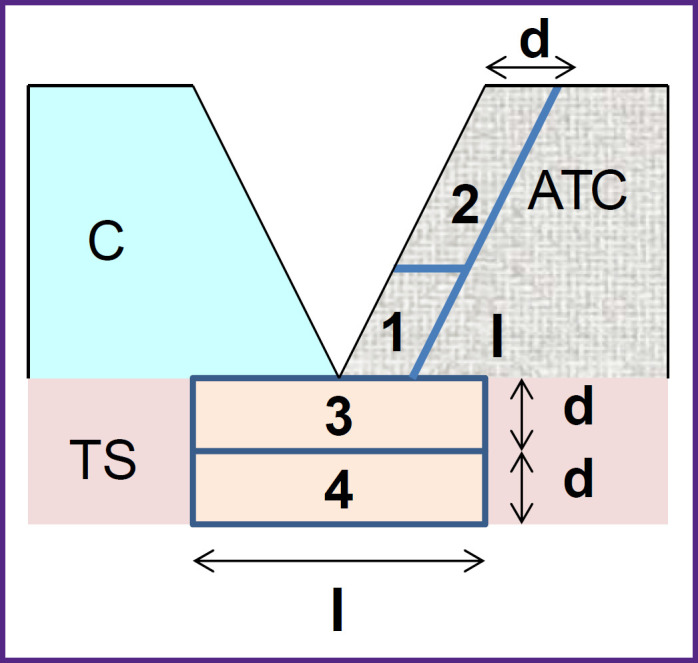
Location of the intact cartilage (C), analogue of the tissue-engineered construction (ATC), trypsinized substrate (TS), and areas for thermal analysis: *1* — part soldered to the first layer of albumin, d=0.4±0.1 mm, l=0.7±0.1 mm; *2* — part soldered to the second/third layer of albumin, d=0.4±0.1 mm, l=1.3±0.1 mm; *3* — upper part of the substrate, d=0.3±0.1 mm, l=1.4±0.2 mm; *4* — lower part of the substrate, d=0.3±0.1 mm, l=1.4±0.2 mm

The basic cartilage and ATCs were cut into strips of 4.0±0.4 mm wide, 1.9±0.3 mm thick, and 14–18 mm long. Using a special knife, an angular cut was made on one side of each strip with an angle of 15°. Then, one strip of each material was selected and connected on a hydrophobic surface. To assess substrate degradation, in some experiments, strips were put on a trypsinized cartilage of 0.6±0.2 mm thick (Figure 1).

A 25% solution of bovine serum albumin (A7030-100G; Sigma-Aldrich, USA) was used as the solder.

### Experimental technique

The gap between the ATC and the intact cartilage was filled with 8 μl of 25% albumin solution. The first dose of albumin partially leaked under the sample and did not completely fill the gap. After laser exposure, the first layer created a substrate on which the next dose of albumin (5 μl) stayed without leakage. After a repeated laser exposure, a second layer was formed. In case of radiation with λ=1.68 μm, two layers of albumin were sufficient to completely close the gap. In case of using radiation with λ=1.56 μm, a third layer of albumin was applied, which was exposed to laser.

The experiments were conducted using two sources of CW laser radiation: an erbium-doped fiber laser with λ=1.56 μm and a fiber Raman laser with λ=1.68 μm (both produced by IRE-Polus, Russia). The radiation power was monitored with a UP12-H power meter (Gentec Electro-Optics, Canada). Radiation with λ=1.56 μm and λ=1.68 μm was supplied through an optical quartz fiber with a 0.22 numerical aperture and a core diameter of 400 and 600 μm, respectively.

**Figure 2.**
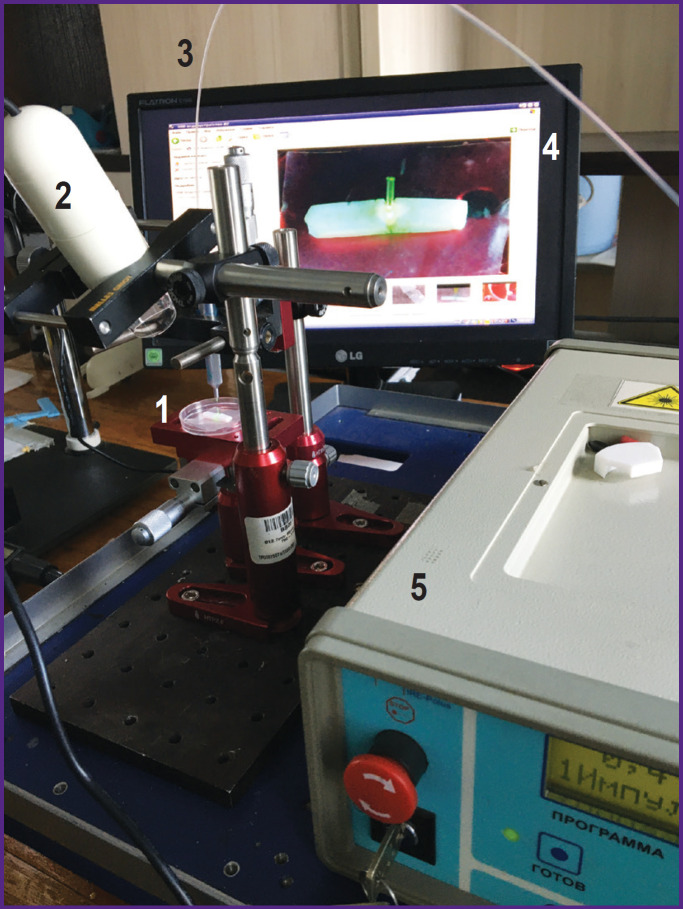
Experimental setup for laser soldering: *1* — sample; *2* — digital USB microscope; *3* — optical fiber; *4* — monitor with the image of the studied object; *5* — laser

The sample on the hydrophobic surface or substrate was placed horizontally, and the fiber was placed above it. All movements were made using micrometric screws. The distance from the fiber end to the irradiated surface ranged from 1 to 3 mm for different albumin layers. The laser exposure was targeted and continued for 10 s, the distance between the centers of the laser spots was equal to the radius of the laser spot. The process of irradiation of the soldering area was monitored using a Dino-Lite Premier digital USB microscope (AnMo Electronics Corporation, Taiwan). The setup is shown in Figure 2. For each exposure mode, 12 to 15 samples were obtained.

After laser irradiation, the thickness and width of the samples in the soldering area were determined and mechanical tests on the samples were conducted. Then, for each exposure mode, the areas immediately adjacent to the soldering area were isolated from three ATC samples, and collagen retention in them was examined by means of differential scanning calorimetry (DSC). The areas of the soldered ATCs and trypsinized substrate subjected to the DSC analysis are schematically shown in Figure 1.

In addition, mechanical tests were conducted for one of the modes and three samples after keeping the samples in a 0.15 M of NaCl solution for 24 h.

In a separate experiment, the dynamics of the temperature field was recorded. The setup was similar to that shown in Figure 2; however, a FLIR A655sc thermal imager camera with a FOL25 lens (FLIR Systems Inc., USA) was used instead of a microscope. The frame frequency was 3.125 Hz. The spatial resolution was 10 px/mm. Using the FLIR Research IR Max software, thermograms were processed, and the following parameters were determined: the maximum temperature dynamics on the surface, the average heating temperature in the laser exposure area, and the temperature profile along the diameter of the heating area.

### Thermal analysis

The thermal behavior of the samples was studied using a differential scanning calorimeter (DSC 204 Phoenix model; Netzsch, Germany). Samples with the weight of 1.5–3.0 mg were sealed in the standard 20 μl aluminum crucibles. When albumin solution was used, 10 μl of this solution was placed in the crucible. The initial and final temperatures were 20 and 90°C, respectively, and the heating rate was 10 K/min. The fraction of intact protein (β) in the sample was determined by the decrease in the thermal effect Δ*H* of denaturation in experimental samples: β=Δ*H*/Δ*H_d_*, where Δ*H_d_* and Δ*H* are the thermal effect of collagen denaturation in samples from the soldering areas 1–4 and the ATC without laser exposure.

### Mechanical tests

Mechanical tests were conducted using a multifunctional desktop testing machine EZ Test (EZ-SX model; Shimadzu, Japan) at the room temperature under uniaxial tensile conditions. During stretching at a rate of 0.2 mm/s, the force–displacement data were automatically recorded, and then the tensile strength was calculated.

### Statistical data processing

Experimental data from the mechanical tests were processed using the OriginPro 2015 software package (OriginLab Corporation, USA). The correspondence of the tensile strength values sample to Gaussian distribution was assessed using the Shapiro–Wilk test. In case of Gaussian distribution, the mean (M) and standard deviation (SD) were determined. The difference between the mean values of separate samples for specimen after exposure to radiation with λ=1.56 μm and λ=1.68 μm was assessed using a two-sample unpaired Student’s t-test. The dispersion test required for application of the t-test was conducted using the Fisher’s ratio test. It should be noted that the use of the Mann–Whitney U-test to determine differences in the mean values seemed inappropriate to the authors due to some coinciding values in the sample data.

If the distribution differed from Gaussian distribution, as well as in cases where the sample size did not exceed four units, the data were shown as a range of final values (min–max).

## Results

**Figure 3.**
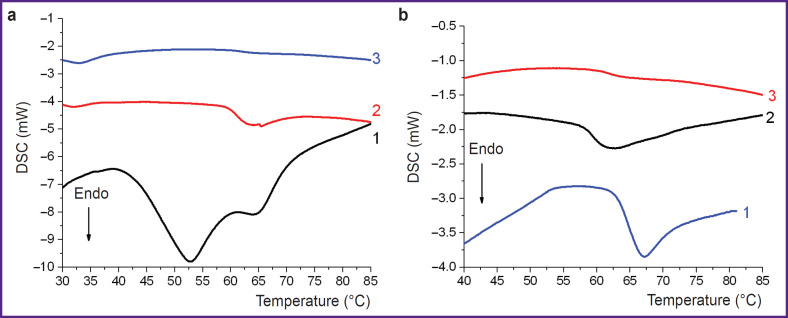
Examples of DSC thermograms showing the number of intact protein macromolecules that denaturate being heated in a calorimeter: albumin (a) initial (*curve 1*); in the solder material after exposure to radiation with λ=1.56 μm, power density of 1.20/1.10/ 0.85 W/mm^2^ (*curve 2*); in the solder material after exposure to radiation with λ=1.56 μm, power density of 1.77/1.34/0.96 W/mm^2^ (*curve 3*); collagen (b) in the ATC material (*curve 1*); in area 1 of the ATC after exposure to radiation with λ=1.68 μm and power density of 0.70/0.80 W/mm^2^ (*curve 2*); in area 2 of the ATC after exposure to radiation with λ=1.68 μm and power density of 0.70/ 0.80 W/mm^2^ (*curve 3*); the arrow indicates the direction of the signal change due to endothermic process in the system

Thermal analysis of the 25% albumin solution showed that denaturation of this protein occurs in two stages in the range of 40–70°C (Figure 3 (a), *curve 1*) with a thermal effect of 15.6±0.5 J/g. Collagen in the ATC material denatured in the range of 61–69°C with a maximum of 65±1°C (Figure 3 (b), *curve 1*). The thermal effect of denaturation Δ*H_d_* was 10±1 J/g.

**Figure 4.**
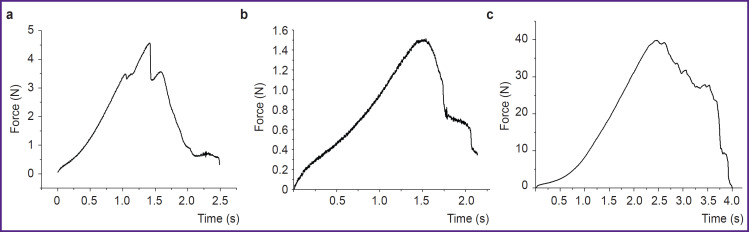
Typical examples of the results of mechanical tests of the soldering samples quality (λ=1.68 μm) using the multifunctional testing machine: (a) soldering of the intact cartilage and the ATC; (b) soldering of the intact cartilage and the ATC after hydration; (c) intact cartilage

At the initial stage of the experiment dedicated to laser soldering of samples, a minimum power density (I_min_) was established for exposure on the first layer of albumin, below which tissues were not soldered. Some albumin in the solder remained intact after such exposure (Figure 3 (a), *curve 2*). After laser exposure with a power density equal to I_min_, the final result of tissue soldering was unpredictable. Some samples fell apart even with careful manipulation, making mechanical tests impossible. When the power density increased above I_min_, all solder albumin was denatured under laser exposure (Figure 3 (a), *curve 3*), whereas the intact cartilage–ATC system was stable. Mechanical tests confirmed that the soldered parts behaved as an integral whole (Figure 4 (a), (b)); the tensile strength σ reached significant values (Table 1), although these values were less than σ=5.8 MPa for the intact sample (Figure 4 (c)).

**T a b l e 1:** Parameters of laser exposure and sample characteristics

I, in the 1/2/3 albumin layer (W/mm^2^)	Tensile strength σ (MPa), min-max, M±SD	Fraction of intact collagen β in areas (%)			
		1	2	3	4
λ=*156 μm*					
1.41/1.34/0.96	0–0.2	50–60	>95	80–90	100
1.77/1.34/0.96	0.7±0.2*	30–40	80–90	60–70	100
*λ=1.68 μm*					
0.55/0.80	0–0.15	10–15	70–90	20–30	80–100
0.7/0.8	0.85±0.20*	<10	60–80	10–20	60–80

* average σ values had no statictically significant differences.

Table 1 provides the characteristics of laser exposure at I=I_min_ and I>I_min_, as well as the results of mechanical tests of the soldered samples. One should note that in samples soldered with laser radiation (λ=1.68 μm, power density — 0.7 W/mm^2^), there was the following effect seen: a decrease in tensile strength on the surface of the first albumin layer by 1.5–2.5 times after hydration (Figure 4 (b)).

Thermal analysis of the ATCs and trypsinized substrate in the areas of soldering (see Figure 2) showed that there was a noticeable degradation of the collagen scaffold adjacent to the first layer of albumin (see Table 1).

**Figure 5.**
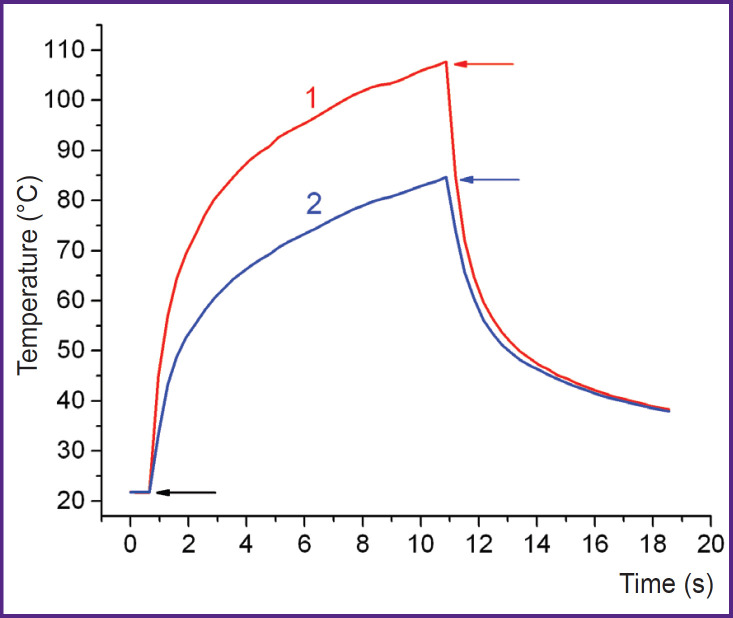
Typical dynamics of the maximum (1) and average (2) temperatures using the example of the exposure to laser radiation with the wavelength of 1.68 μm on the second layer of albumin Arrows indicate the start and end of laser irradiation

**Figure 6.**
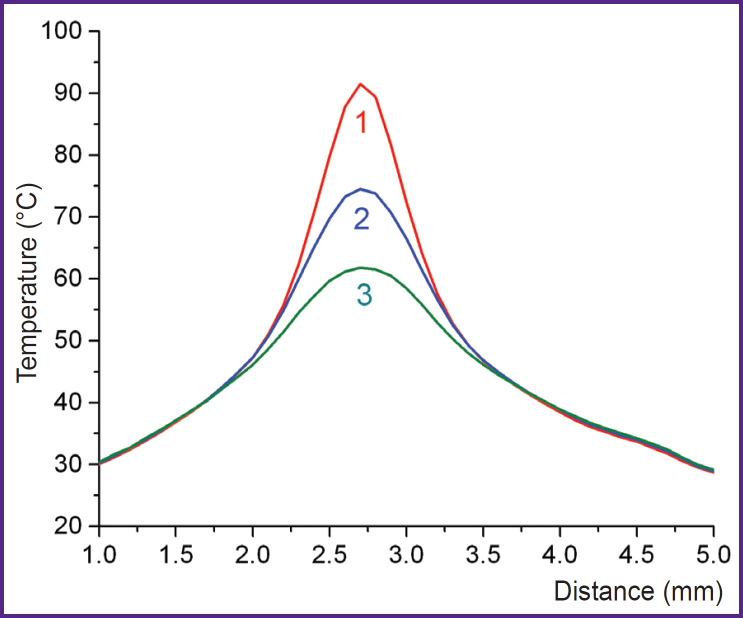
Typical temperature distributions along the diameter of the heated area using the example of the exposure to laser radiation with the wavelength of 1.68 μm on the first layer of albumin: *1* — at the moment of switching off the laser, 2 and *3* — 0.32 and 0.64 s after switching off the laser, respectively

Analysis of the dynamics of the maximum T_max_ and average T_av_ temperatures on the albumin surface in the exposure area showed that the temperature increased during the laser exposure and decreased quite quickly after the laser shut down (Figure 5), here the heated area did not expand (Figure 6). T_max_ and T_av_ values at the laser shut down are provided in Table 2.

**T a b l e 2:** Temperature characteristics of the laser exposure on albumin surface

λ=1.56 μm			λ=1.68 μm		
I (W/mm^2^)	T_max_ (°C)	T_av_ (°C)	I (W/mm^2^)	T_max_ (°C)	T_av_ (°C)
1.41 (layer 1)	73	62	0.55 (layer 1)	80	64
1.77 (layer 1)	11 4	96	0.70 (layer 1)	92	80
1.34 (layer 2)	11 6	95	0.80 (layer 2)	107	85
0.96 (layer 3)	11 0	102			

## Discussion

With the concentration used herein (25%), albumin denaturation proceeded in two stages. The shape of the DSC curve, as well as Δ*H_d_*, corresponded to the literature data [18]. Denaturation was complete at 70°C. When the entire volume of albumin was not sufficiently heated, the protein denaturation was not complete. When the laser power density was below I_min_, the average temperature of the whole laser spot area was below 65°C. Hence, albumin denaturation followed by coagulation and “solidification” of the solution was a requisite condition for effective tissue soldering with albumin. This assumption about the soldering mechanism was put forward by other researchers [3, 19], and we confirmed this experimentally.

In this study, the tensile strength σ was slightly less than 1 MPa (see Table 1), which is close to the values obtained both for cartilage–cartilage soldering [5, 7] and for other collagen-containing tissues [2, 7, 15, 19, 20]. In study [7], the value σ=1.9 MPa was obtained for soldered tracheal cartilage using albumin solder with carbon particles added, which increased the absorption of laser radiation. Eventually, variations in the chemical composition of the solder and the radiation power (solder temperature) ensured an almost twofold increase in σ. As the authors used the minimum possible power, an increase in power was inevitably accompanied by an increase in both the maximum and average temperatures of albumin, including in the layer adjacent to the tissue. In turn, this led to additional degradation of these tissue areas (see Table 1). Actually, soldering of the tissue-engineered construction (graft) and cartilage was only a temporary fixation of the graft. The resulting strength was sufficient for this purpose [5]. It should be noted that albumin leaking under the construction and its subsequent denaturation during the replacement of a defect directly in the articular cartilage resulted in additional strengthening of the system [5]. Moreover, this configuration with a graft and a prepared cavity in the articular cartilage prevented separation of the solder from the tissue during hydration. For instance, when the soldered strips were kept in an aqueous solution for more than a day, the solder gradually detached from the tissue and, thus, the strength of the samples decreased [2, 21]. We saw a noticeable decrease in strength when the samples were hydrated for 24 h. At the same time, when a graft customized in size was solded into the cartilage cavity, hydration was not accompanied by a decrease in the system strength [5], as the contact area of the soldering locus with the liquid was significantly less than the total area of the solder.

An important aspect of using laser soldering in a living system is keeping tissues in a functional state. Researchers who determined structural changes in soldered tissues mentioned noticeable damage in the tissue adjacent to the soldering locus [22], particularly, cell death [5], necrosis and coagulation [6], as well as changes in collagen structure [23]. At that, soldering was conducted with a sensitizer in the solder, that is the tissue did not absorb the laser radiation. Nevertheless, thermal degradation of the tissue happened due to heat dissipation from the exposed area. The degradation area practically coincided with the size of the laser spot, and the depth was significant [5, 6]. The radiation herein was absorbed by water, thus heating both the solder and the adjacent tissues if the depth of radiation absorption was sufficient. Eventually, the collagen network of the ATC had the maximum damage in areas 1 and 3 (see Figure 1), where radiation penetrated the tissue (see Table 1). It is important to note that the tissue was not heated outside the exposure area due to thermal conductivity (see Figure 6), and the damage was significantly lower in areas 2 and 4. Moreover, for radiation with λ=1.68 μm, the proportion of intact collagen preserved was significantly lower than that for radiation with λ=1.56 μm. Obviously, this was due to the typical penetration depth of radiation δ, which for cartilage tissue was 1.35–1.45 mm at λ=1.68 μm, which is almost two times higher than δ=0.86 mm for λ=1.56 μm [24]. On the other hand, at a higher value of δ (due to a lower effective absorption coefficient [24]), the temperature decrease with depth was less significant, and the volume was heated more homogeniously. This was proved by the lower temperature values recorded on the surface of the sample in the modes of effective soldering by radiation with λ=1.68 μm compared to λ=1.56 μm (see Table 2). As a result, heating of the entire solder volume at λ=1.68 μm required less radiation power I than at λ=1.56 μm. With I=0.7–0.8 W, the temperature of the heated volume was ~70–80°C, which correlated with the optimal laser soldering temperature obtained from systematic studies of this phenomenon [2, 19, 20]. In addition, the use of the laser radiation with λ=1.68 μm allowed to limit albumin to two layers in contrast to radiation with λ=1.56 μm, when three layers of the solder were required.

As for a certain tissue degradation, this phenomenon is inevitable both with sensitized and direct heating. However, low-volume tissue degradation is not a key obstacle to the use of laser soldering *in vivo*, as albumin is a therapeutic agent [25] and effectively promotes regeneration after laser soldering [6].

## Conclusion

Laser soldering of a tissue-engineered construction analogue to replace an osteochondral defect and intact cartilage tissue can be effectively conducted using radiation with wavelengths of 1.56 μm and 1.68 μm, absorbed both by the solder and by the tissue. The parameters of the laser exposure provided such a space-temporal temperature distribution that the area filled with an albumin solution was heated to the temperature of albumin denaturation. At the irradiated tissue edges, the temperature was lower, and the collagen network degradation occurred in a local area adjacent to the bottom of the tissue solder. To minimize the area of degradation, one should reduce the diameter of the laser spot so that its size, if possible, matches the area where the solder filled the cavity between the construction and the intact cartilage. It should be noted that the profile of the tissue-engineered construction edge and the width of the cavity can be slightly varied because the leakage of the albumin solution was limited by the integrity of the intact part of the cartilage and the bone substrate. Due to that, it was possible to reduce both the number of solder layers and the degree of degradation of the soldered tissues, which should be considered in the following studies.
